# Discrimination of Prion Strain Targeting in the Central Nervous System via Reactive Astrocyte Heterogeneity in CD44 Expression

**DOI:** 10.3389/fncel.2019.00411

**Published:** 2019-09-10

**Authors:** Barry M. Bradford, Christianus A. W. Wijaya, Neil A. Mabbott

**Affiliations:** The Roslin Institute and R(D)SVS, The University of Edinburgh, Edinburgh, United Kingdom

**Keywords:** prion, transmissible spongiform encephalopathy, neurodegeneration, neuroinflammation, astrocyte, CD44

## Abstract

Prion diseases or transmissible spongiform encephalopathies are fatal, progressive, neurodegenerative, protein-misfolding disorders. Prion diseases may arise spontaneously, be inherited genetically or be acquired by infection and affect a variety of mammalian species including humans. Prion infections in the central nervous system (CNS) cause extensive neuropathology, including abnormal accumulations of misfolded host prion protein, vacuolar change resulting in sponge-like (spongiform) appearance of CNS tissue, neurodegeneration and reactive glial responses. Many different prion agent strains exist and these can differ based on disease duration, clinical signs and the targeting and distribution of the neuropathology in distinct brain areas. Reactive astrocytes are a prominent feature in the prion disease affected CNS as revealed by distinct morphological changes and upregulation of glial fibrillary acidic protein (GFAP). The CD44 antigen is a transmembrane glycoprotein involved in cell-cell interactions, cell adhesion and migration. Here we show that CD44 is also highly expressed in a subset of reactive astrocytes in regions of the CNS targeted by prions. Astrocyte heterogeneity revealed by differential CD44 upregulation occurs coincident with the earliest neuropathological changes during the pre-clinical phase of disease, and is not affected by the route of infection. The expression and distribution of CD44 was compared in brains from a large collection of 15 distinct prion agent strains transmitted to mice of different prion protein (*Prnp*) genotype backgrounds. Our data show that the pattern of CD44 upregulation observed in the hippocampus in each prion agent strain and host *Prnp* genotype combination was unique. Many mouse-adapted prion strains and hosts have previously been characterized based on the pattern of the distribution of the spongiform pathology or the misfolded PrP deposition within the brain. Our data show that CD44 expression also provides a reliable discriminatory marker of prion infection with a greater dynamic range than misfolded prion protein deposition, aiding strain identification. Together, our data reveal CD44 as a novel marker to detect reactive astrocyte heterogeneity during CNS prion disease and for enhanced identification of distinct prion agent strains.

## Introduction

Prion diseases, or transmissible spongiform encephalopathies, are a unique group of infectious, sub-acute, neurodegenerative disorders. These disease have been identified in several mammalian species and include scrapie in sheep and goats, bovine spongiform encephalopathy (BSE) in cattle, chronic wasting disease (CWD) in cervids and Creutzfeldt-Jakob disease (CJD) in humans. During prion disease, abnormally folded isoforms (PrP^Sc^) of the host-encoded cellular prion protein (PrP^C^) accumulate and aggregate in affected tissues (Prusiner, 1982). The infectious prion is considered to constitute almost entirely of PrP^Sc^ implying that prions are infectious proteins. Prion disease within the central nervous system (CNS) causes extensive neuropathology and neurodegeneration. This characteristically includes neuronal vacuolation resulting in a spongiform (sponge-like) appearance of brain tissue in histopathological specimens (Fraser and Dickinson, 1967) and progressive loss of dendritic spines (Fang et al., 2016), synapses (Jeffrey et al., 2000), axons (Jeffrey et al., 1995) and neuronal cell bodies (Giese et al., 1995). These histopathological signs are also accompanied by activation of the astrocytes and microglia (Kercher et al., 2007).

Many distinct prion agent strains have been identified and characterized by transmission into laboratory mice (Boyle et al., 2017). Once adapted to their new host environment, these prion strains can be stably maintained by passage within that same host species, resulting in reproducible disease incubation periods, clinical presentation and targeting of prion neuropathology (Dickinson and Fraser, 1977). As well as conservation of biological properties, prion strains also have preserved biochemical properties, which has led to the hypothesis that the distinct conformational state of the misfolded prion protein encodes this diversity (Safar et al., 1998; Peretz et al., 2001, 2002; Arima et al., 2005). The diversity in the targeting of neuropathological changes within the CNS resulting from different prion strains has been key to facilitating strain identification (Fraser and Dickinson, 1967, 1968, 1973; Bruce et al., 1989, 1991; Fraser, 1993; van Keulen et al., 2015; Boyle et al., 2017).

Reactive astrocytes are a prominent feature in the prion disease affected brain but whether they play a role in the development of, or protection from, the neurodegeneration during prion disease is uncertain. Both neurons and astrocytes can facilitate prion agent replication (Diedrich et al., 1991; Race et al., 1995; Raeber et al., 1997; Kovács et al., 2005; Sarasa et al., 2012; Krejciova et al., 2017) and induce microglial recruitment (Marella and Chabry, 2004) at early stages of prion disease neuropathogenesis. Indeed expression of the prion protein gene (*Prnp*) in astrocytes alone is sufficient to support prion infection in the brains of infected mice (Raeber et al., 1997). Data from an *in vitro* study have suggested that the reactive astrocytes may also play a role in the recruitment of microglia toward regions of the brain affected by prions (Marella and Chabry, 2004). To-date, analyses of the astrocytic response during prion infection have predominantly focused on the immunohistochemical detection of upregulated expression of the intermediate filament glial fibrillary acidic protein (GFAP) and morphological changes to the astrocyte cytoskeleton (Georgsson et al., 1993; Monzon et al., 2018). However, independent studies have shown that the activation status of the reactive astrocytes is highly heterogeneous (Zamanian et al., 2012), and can be broadly categorized into neurotoxic ‘A1’ or neuroprotective ‘A2’ phenotypes based on functional and transcriptional characteristics (Liddelow et al., 2017). Whether CNS prion infections also lead to the development of neurotoxic or neuroprotective phenotypes in astrocytes, and whether this differs amongst different prion agent strains is not known.

Transcriptional analyses have shown that expression of the adhesion molecule CD44 is significantly elevated in reactive astrocytes induced by a range of pro-inflammatory stimuli (Liddelow et al., 2017). Furthermore, the expression of CD44 expression was significantly elevated in neurotoxic A1 astrocytes when compared to the A2 astrocytes with a neuroprotective phenotype. Therefore, in the current study we used the immunohistochemical analysis of CD44 expression to characterize the heterogeneity of the reactive astrocyte response in the brains of mice infected with a large range of distinct prion agent strains. We show that in the brains of mice infected with prions, strong astrocyte-associated CD44 expression was detected as early as halfway through the disease incubation period and concurrent with some of the earliest neuropathological changes. Data from prion disease transmission to mice have revealed the discriminatory properties and limitations of disease-specific vacuolation or PrP^d^ in identifying prion disease strain and host differences. Furthermore, not all prion diseases are transmissible to laboratory mice, and for novel natural prion disease cases the incubation period of disease is often unknown. The identification of a novel marker of prion disease that can discriminate prion strains in different host *Prnp* genotypes irrespective of survival time or route of infection could prove useful in understanding the variety of strains in natural prion disease cases both in humans and animals. Our data from the analysis of a large collection brains from mice infected with 15 distinct prion agent strains suggest CD44 expression fulfils these criteria and can be used as a novel marker to detect reactive astrocyte heterogeneity during CNS prion disease.

## Materials and Methods

### Animals

C57BL/Dk and VM/Dk mice were bred and housed under specific pathogen-free conditions with a 12:12 h light:dark cycle. Food and water were provided *ad libitum*. Necessary approvals for the mouse experiments described in this study were obtained from the Institute for Animal Health Neuropathogenesis Unit Ethical Review Committee, the University of Edinburgh Ethical Review Committee, and the UK Home Office.

### Prion Infection

Groups of C57BL/Dk, VM/Dk and F1 cross offspring termed “CVF1” mice were injected intracerebrally (IC) with 20 μl of a 1% (weight/volume) brain homogenate prepared from mice terminally infected with prions. For intraperitoneal (IP) or intravenous (IV) infection the mice were injected with the same dose and volume of prions into the peritoneal cavity or tail vein, respectively. For oral infection mice were individually housed overnight in bedding-free cages with a single food pellet dosed with 50 μl of a 1% (weight/volume) brain homogenate. The mice were maintained in these cages until the food pellet was fully consumed, upon which they were then returned to standard group caging. All mice were coded and assessed by independent technicians at weekly intervals for the clinical signs of prion disease. Mice were scored as “unaffected,” “possibly affected” and “definitely affected” using standard criteria (Brown et al., 2012) and culled at a standard clinical end-point (maximally on their 3rd clinical assessment of definitely affected). Mean disease survival times were calculated as the interval between infection with prions and the detection of positive clinical signs of terminal prion disease.

### Neuropathological Analysis

Clinical prion disease diagnoses were confirmed by histopathological assessment of vacuolation (spongiform pathology) in the brain. Brains were codified at post-mortem and fixed in 10% formal saline, trimmed into 5 routine coronal slices based on external features, processed and embedded in paraffin wax. Sections were cut at 6 μm thickness, stained with hematoxylin & eosin and scored for spongiform vacuolar degeneration by blinded assessment as described previously (Fraser and Dickinson, 1967). Sections were scored by an independent scientist for the presence and severity (scale 0–5) of prion-disease-specific vacuolation in nine gray matter and three white matter brain areas: G1, dorsal medulla; G2, cerebellar cortex; G3, superior colliculus; G4, hypothalamus; G5, medial thalamus; G6, hippocampus; G7, septum; G8, cerebral cortex; G9, forebrain cerebral cortex; W1, cerebellar white matter; W2, midbrain white matter; W3, cerebral peduncle.

### Immunohistochemistry (IHC)

Sections (6 μm) were cut on a HM325 Rotary Microtome (Thermo Fisher Scientific, Runcorn, United Kingdom) and mounted on Superfrost Plus slides (Menzel-Glaser GmbH, Braunschweig, Germany). Before immunostaining the sections were deparaffinised using an XL Autostainer (Leica Biosystems, Newcastle upon Tyne, United Kingdom) and pre-treated by autoclaving (15 min at 121°C) in Target Retrieval Solution pH 6.0 (DAKO UK Ltd., Ely, United Kingdom). For the detection of PrP sections were then immersed in formic acid (98%) for 5 min, and subsequently immunostained with anti-PrP-specific mAb [Clone BH1] (Morales et al., 2007). To detect astrocytes the sections were immunostained with rabbit anti-GFAP (DAKO), biotinylated anti-mouse/human CD44 [Clone: IM7] (Biolegend, London, United Kingdom) or anti CD44var6 [Clone 9A4] (eBioscience, Ltd., Hatfield, United Kingdom). To detect microglia the sections were immunostained with anti-Iba1 polyclonal antibody (AIF1; Wako Chemicals GmbH, Neuss, Germany). Following the addition of primary antibodies, biotin-conjugated species-specific secondary antibodies (Jackson ImmunoResearch Europe Ltd., Ely, United Kingdom) were applied. Biotinylated antibidies were detected using horse radish peroxidase-conjugated to the avidin-biotin complex [HRP-ABC] kit (Vector Laboratories, Peterborough, United Kingdom) and visualized with 3,3’-diaminobenzidine [DAB] (Merck KGaA, Darmstadt, Germany) and counterstained with hematoxylin. For dual CD44/GFAP staining, CD44 was detected using the HRP substrate Vector NovaRed (Vector Laboratories, Peterborough, United Kingdom) and GFAP detected with alkaline phosphatase conjugated anti-rabbit antibody (Jackson Immunoresearch) and 5-bromo-4-chloro-3-indolyl phosphate/nitro blue tetrazolium (BCIP/NBT) substrate (Merck). Sections were viewed using an Eclipse Ni-E microscope (Nikon Instruments Europe BV, Amsterdam, Netherlands) with Zen software (Carl Zeiss Ltd. Cambridge, United Kingdom).

### Immunofluorescence

For immunofluorescence analysis, goat anti-rabbit iFluora594 (AAT Bioquest, Sunnyvale, United States) was applied following anti-GFAP immunostaining, and goat anti-mouse Alexaflluor647^TM^ (Life Technologies Ltd., Inchinnan, United Kingdom) was applied following anti-PrP (clone BH1) immunostaining. For CD44 and CD44v6 staining procedures were performed as above and visualized using the HRP substrate Tyramide iFluora488 (AAT Bioquest). Sections were mounted using fluorescent mounting medium (DAKO) before analysis. Images were captured using a confocal laser scanning LSM710 microscope with Zen software (Zeiss).

### Thioflavin Staining of Amyloid

Paraffin-embedded sections as above were dewaxed and rehydrated, incubated in 1% thioflavin-S (Merck KGaA) for 5 min, differentiated in 70% industrial denatured alcohol for 3 × 1 min and washed in distilled water. Slides were mounted using fluorescent mounting medium (DAKO) and images captured using a confocal laser scanning LSM710 microscope with Zen software (Zeiss).

### Image Analysis

For morphometric analysis, coded IHC sections and images were analyzed using ImageJ software^1^ by blinded assessment as described (Inman et al., 2005). Images were captured from a minimum of *N* = 6 mice/group. To visualize staining intensity the deconvoluted DAB channel was false-colored using an inverted 16 color lookup table [LUT] representing variance in pixel intensity (see calibration bar in each figure). Thus, each image was color deconvoluted using the vector H-DAB and the percentage area coverage of each IHC marker in each of the 9 gray matter areas was then measured. Data are expressed as mean ± SEM from a minimum of 72 images/profile.

### Statistical Analysis

Survival periods, vacuolation scores and percentage area coverage of IHC staining are presented as mean ± SEM from *N* = 4–31 mice/group. Pearson correlation coefficients were calculated from *N* = 6 mice/group. Survival time and image analysis quantification data were compared using paired *T*-tests. Statistical analyses were performed using Minitab 17 software (Minitab Ltd., Coventry, United Kingdom).

## Results

### Comparison of Survival Times and Neuropathology in a Large Collection of Distinct Mouse-Adapted Prion Agent Strains

The identification or definition of distinct prion strains from various sources has been well characterized by their transmission into laboratory mice (Boyle et al., 2017). These analyses show that distinct prion agent strains can differ significantly in the duration of the survival times and the targeting of the neuropathology (spongiform pathology) in the brain. Therefore, in the current study we compiled a large collection of brains from mice infected with 15 distinct and defined mouse-adapted strains and used it to compare glial cell responses during CNS prion disease. Full details of all the prion agent strains used and the recipient mouse strain backgrounds are provided in Table 1.

**TABLE 1:** Prion agent strains analyzed in this study.

**Strain**	**Disease**	**Origin**	**Passage**	**Passage/line**	**References**
ME7	Natural sheep scrapie	Suffolk sheep	9^th^	C57Bl/Dk	Zlotnik and Rennie, 1963
22A	Experimental sheep scrapie SSBP1	Cheviot sheep	10^th^	VM/Dk	Dickinson and Meikle, 1969
22C	Experimental sheep scrapie SSBP1	Cheviot Sheep	8^th^	C57Bl/Dk	Dickinson, 1976
22F	Experimental sheep scrapie SSBP1	Cheviot Sheep	9^th^	C57Bl/Dk	Dickinson, 1976
22L	Experimental sheep scrapie SSBP1	Cheviot Sheep	13^th^	C57Bl/Dk	Dickinson, 1976
79A	Experimental sheep scrapie SSBP1	‘Drowsy’ goat	12^th^	C57Bl/Dk	Dickinson, 1976; Pattison and Millson, 1961
79V	Experimental sheep scrapie SSBP1	‘Drowsy’ goat	5^th^	VM/Dk	Dickinson, 1976; Pattison and Millson, 1961
80A	Experimental sheep scrapie SSBP1	‘Scratching’ goat	5^th^	C57Bl/Dk	Pattison and Millson, 1961
87A	Natural sheep scrapie	Cheviot × Border Leicester sheep	9^th^	C57Bl/Dk	Bruce et al., 1976
87V	Natural sheep scrapie	Cheviot x Border Leicester sheep	9^th^	VM/Dk	Bruce et al., 1976
139A	Experimental sheep scrapie SSBP1	‘Drowsy’ goat Chandler isolate	9^th^	C57Bl/Dk	Chandler, 1961; Pattison and Millson, 1961
221C	Natural sheep scrapie	Halfbreed sheep	4^th^	C57Bl/Dk	Bruce et al., 2002
301C	BSE	Holstein-Fresian cow	7^th^	C57Bl/Dk	Bruce et al., 1994
301V	BSE	Holstein-Fresian cow	7^th^	VM/Dk	Bruce et al., 1994
409V	CWD	Mule deer	5^th^	VM/Dk	Bruce et al., 2002^∗^

*^∗^Confirmed US CWD case material identified circa 1980 provided by Stuart Young and Beth Williams, Wild Animal Disease Center and Department of Pathology, College of Veterinary Medicine and Biomedical Sciences, Colorado State University, Fort Collins, CO, United States (Williams and Young, 1980).*

**FIGURE 1:**
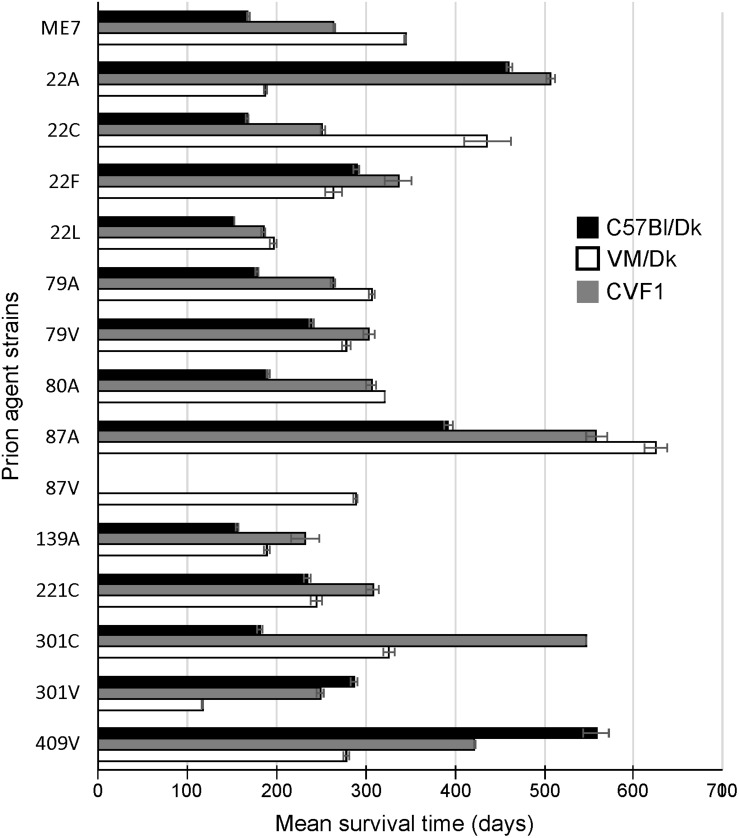
Mean prion disease survival times in laboratory mice infected with a variety of distinct prion agent strains. Groups of C57Bl/Dk (*Prnp*-a), CVF1 (*Prnp*-a/b) or VM/Dk (*Prnp*-b) mice were injected intracerebrally with a variety of distinct prion agent strains (Table 1) and survival times recorded from mice confirmed to have clinical and pathological signs of prion disease. Mean survival times in days ± SEM are displayed for each mouse-passaged prion strain group (*N* = 4–31 mice/group, total = 446 mice). Attempted transmissions of the 87 V strain to C57Bl/Dk or CVF1 mice have not produced positive clinical or pathological signs of prion disease in recipient mice up to at least 600 days post injection.

Prion agent strain characteristics can also be significantly influenced by host *Prnp* genotype (Dickinson and Meikle, 1971). Therefore, in this study we studied brains from C57Bl/Dk mice that have the *Prnp*-a (108L, 189T) genotype, and VM/Dk mice that have a *Prnp*-b (108F, 189V) genotype (Westaway et al., 1987). All mice were injected IC with a 1% dose of brain homogenate and brains were analyzed at the terminal stage of disease. The relative prion disease survival times in the recipient mice are shown in Figure 1. These data show that the order of the relative survival times, particularly in CVF1 mice, can be used to distinguish similar strains such as 79A from 139A for example.

**FIGURE 2:**
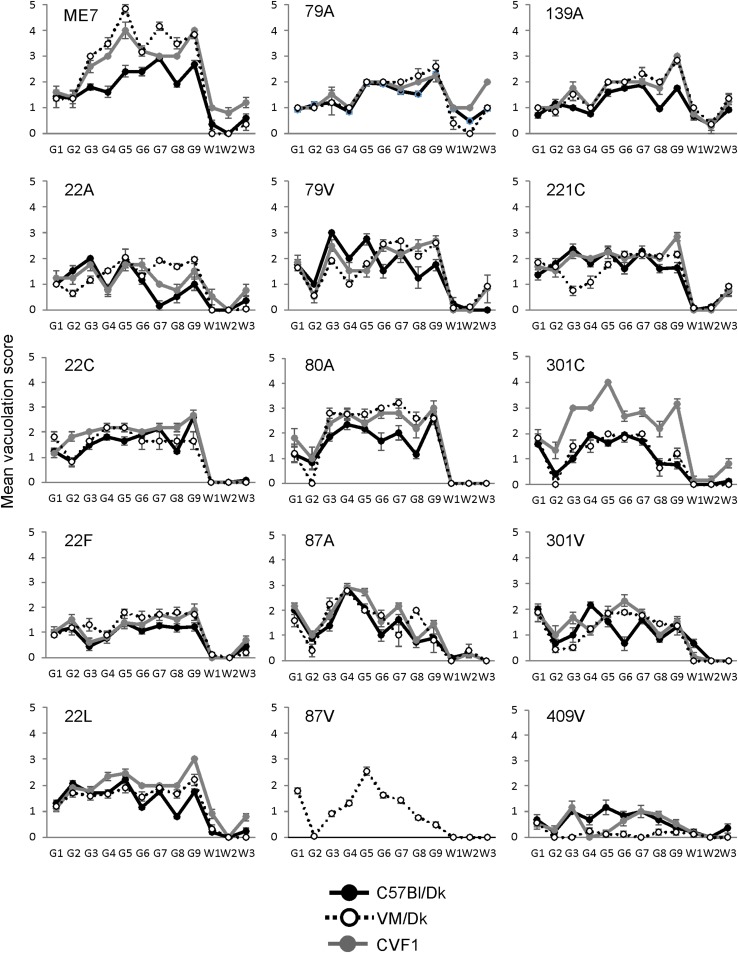
Comparison of the magnitude and distribution of the spongiform pathology in the brains of mice infected with a variety of distinct prion agent strains. Groups of C57Bl/Dk or VM/Dk mice were injected intracerebrally with a variety of distinct prion agent strains (Table 1). Brains were collected at the terminal stage of disease and the magnitude and distribution of the spongiform pathology (vacuolation) scored for each of the following brain regions G1, dorsal medulla; G2, cerebellar cortex; G3, superior colliculus; G4, hypothalamus; G5, medial thalamus; G6, hippocampus; G7, septum; G8, cerebral cortex; G9, forebrain cerebral cortex; W1, cerebellar white matter; W2, midbrain white matter; W3, cerebral peduncle. Data represent mean vacuolation severity scores ± SEM.

The distribution and magnitude of the prion disease-specific spongiform pathology (vacuolation) in nine gray matter regions and three white matter regions of the brains from mice in each group are shown in Figure 2. These data, in combination with the survival time data in Figure 1 have enabled each of these distinct strains to be identified and distinguished from each other in the brains of laboratory mice (Bruce et al., 1997). These data were used here to confirm the nature and identity of these distinct prion agent strains used for the further investigations below.

### PrP^Sc^ Deposition

**FIGURE 3:**
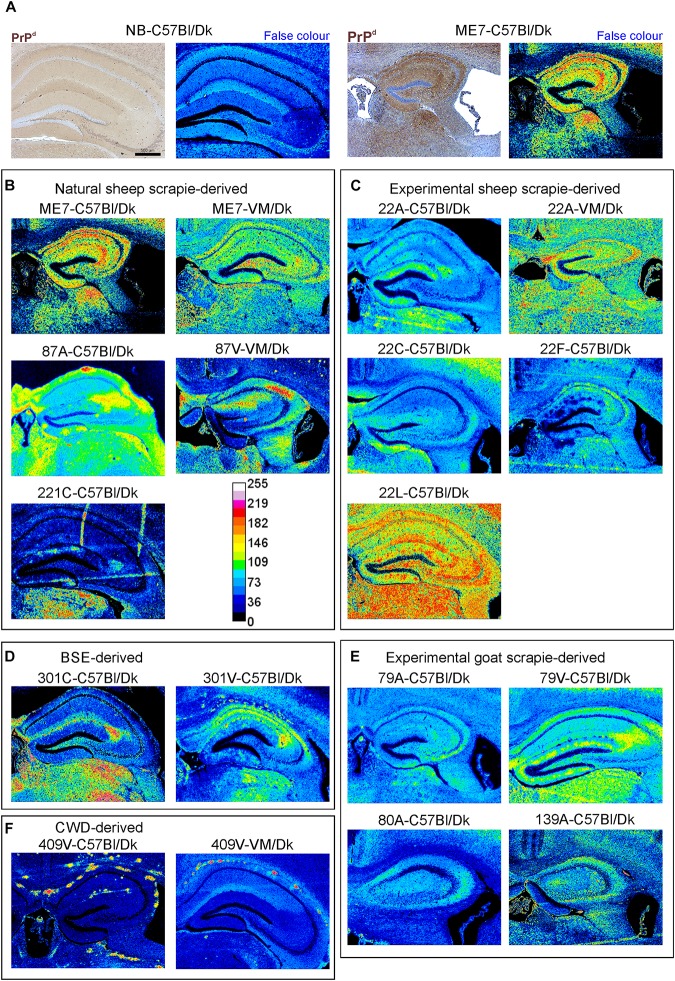
Immunohistopathological comparison of the PrP^d^ immunostaining in the brains of mice infected with a variety of distinct prion agent strains. Groups of C57Bl/Dk or VM/Dk mice were injected intracerebrally with a variety of distinct prion agent strains (Table 1). Brains were collected at the terminal stage of disease and immunostained to detect PrP^d^. Images were then false-colored to compare the signal intensities of the immunostaining between each group. **(A)** Normal brain infected mice display no PrP^d^ compared to mice terminally affected with ME7 scrapie prions. **(B–F)** Comparison of false colored images to show the variation in PrP^d^ + immunostaining between diverse prion strains isolated from a variety of sources. Representative images from 6 mice/group are shown. Scale bars = 500 μm.

The abnormal accumulation of misfolded prion disease-specific PrP (PrP^d^) is a characteristic feature in the brain during CNS prion disease. The pattern of the PrP^d^ deposition within the brains of infected mice has previously been used to help discriminate some prion agent strains, although this was not possible for all strains analyzed (van Keulen et al., 2015). Here, to compare the pattern of PrP^d^ deposition between distinct prion agent strains, terminal brain sections were immunostained using anti-PrP antibody BH1. The resulting images were then false-colored to compare the signal intensities of the immunostaining between strains (Figure 3). In the brains of C57Bl/Dk mice most prion strains could be distinguished by PrP^d^ deposition pattern as shown previously (van Keulen et al., 2015). However, the dynamic range was low due to the diffuse and widespread nature of the PrP^d^ deposition observed for some prion agent strains including 22A, 22C, 22F, 79A, 80A, and 139A (Figures 3C,E). Variance was also minimal between the hippocampal CA2-focussed 87A (Figure 3B) and 79V (Figure 3E) strains, and the intense PrP deposits observed throughout the hippocampus of mice infected with the ME7 (Figure 3B) and 22L prion agent strains (Figure 3C). However, the PrP deposition observed in the brains of mice infected with BSE (Figure 3D) and CWD (Figure 3F) derived prion strains was readily distinguishable from those of scrapie origin (Figures 3B,C,E).

**FIGURE 4:**
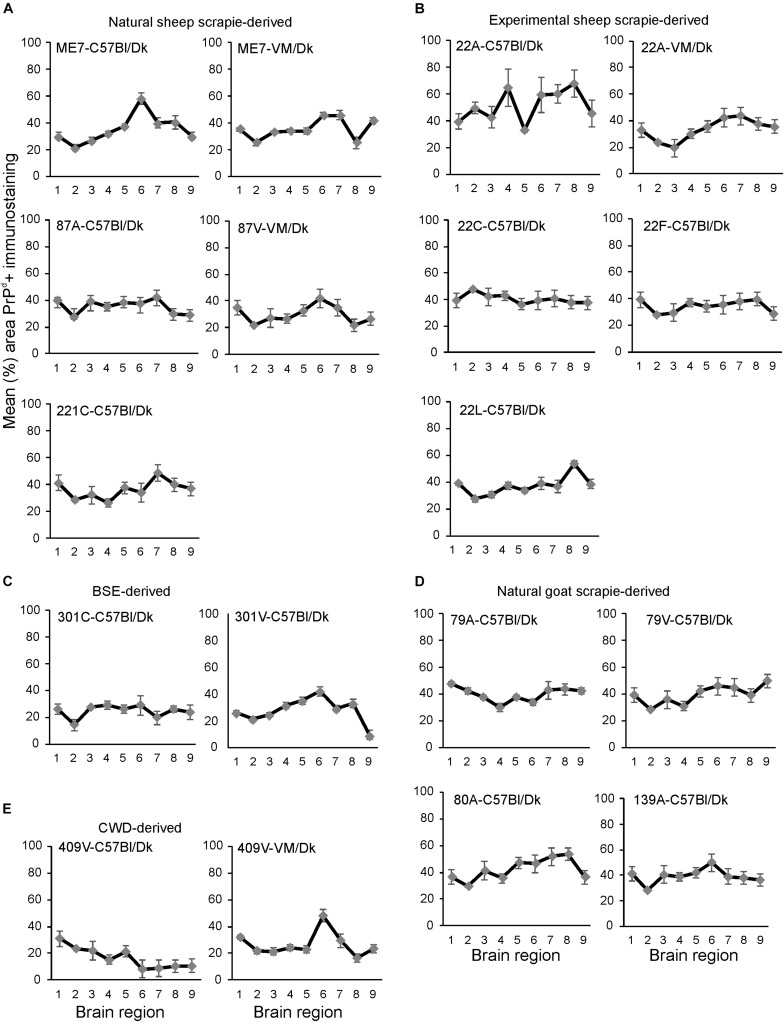
**(A–E)** Comparison of the magnitude and distribution of the PrPd+ immunostaining in the brains of mice infected with a variety of distinct prion agent strains. Groups of C57Bl/Dk or VM/Dk mice were injected intracerebrally with a variety of distinct prion agent strains (Table 1). Brains were collected at the terminal stage of disease and immunostained to detect PrP^d^ and the percentage area coverage compared in each of the following gray matter brain regions G1, dorsal medulla; G2, cerebellar cortex; G3, superior colliculus; G4, hypothalamus; G5, medial thalamus; G6, hippocampus; G7, septum; G8, cerebral cortex; G9, forebrain cerebral cortex. *N* = 6 mice/group. Data represent mean ± SEM, from a minimum of 54 images/group.

Next, the percentage area coverage of PrP^d^+ immunostaining was measured across nine distinct brain gray matter areas (Figure 4). The intensity of the PrP^d^+ immunostaining across these areas was then compared between each prion agent strain by paired *T*-test (Supplementary Table 1). This analysis showed the highly discriminatory nature of PrP^d^ detection in prion agent strain typing and revealed statistically significant differences in 55% (84/153) of comparisons. For example 22A-C57Bl/Dk was almost completely distinguishable by PrP^d^ profile against all other strains, except when compared against 80A-C57Bl/Dk (*P* = 0.052, Supplementary Table 1). Similarly the PrP^d^ profiles for BSE (301C & 301V) and CWD (409V) derived prion agent strains were readily discriminated from most natural and experimental scrapie strains with high levels of significance. However as noted above, this analysis showed that several strains were indistinguishable across many brain regions due to similarities in the amount of PrP^d^ present, as reported previously (van Keulen et al., 2015).

**FIGURE 5:**
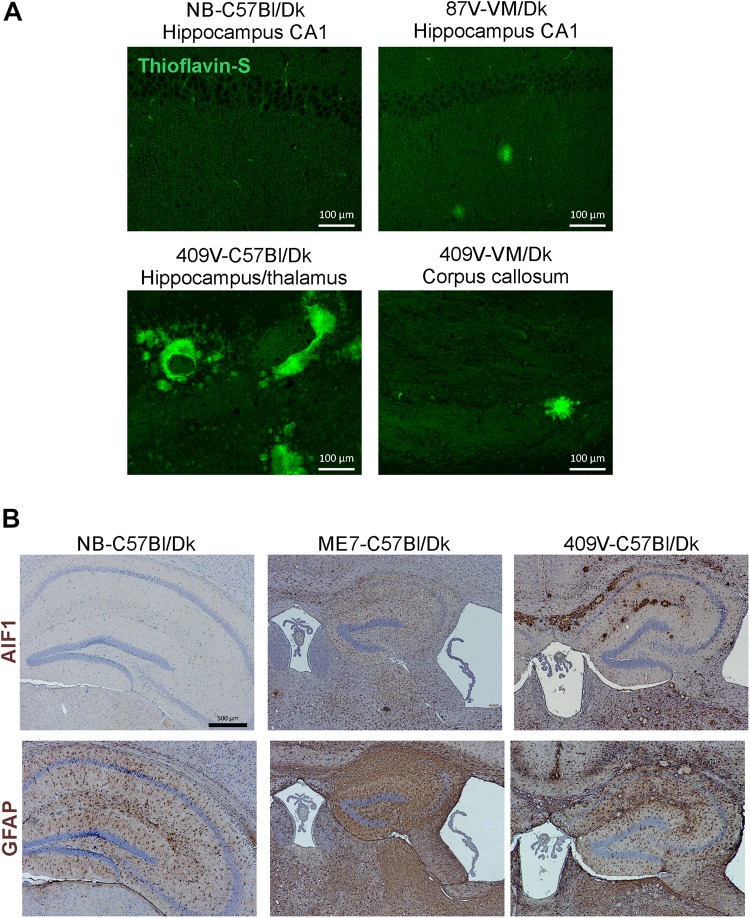
Histopathological detection of amyloid and activated glial cells in the brain following prion infection. **(A)** Thioflavin-S staining of normal brain C57Bl/Dk, 87V-VM/Dk, 409V-C57Bl/Dk and 409V-VM/Dk prion agent strain and host combinations revealed characteristic amyloid deposits in the brains of mice with amyloidal PrP accumulations. Images are representative of *N* = 4 mice/group. Scale bars = 100 μm. **(B)** Immunohistochemical detection of microglia (AIF1+ cells, upper row) and astrocytes (GFAP+ cells, lower row). This analysis showed minimal AIF1+ and GFAP+ immunostaining in the brains of uninfected control mice, whereas expression of these markers was strongly upregulated in brains of mice with terminal prion disease. Representative images *N* = 6 mice/group are shown. Scale bars = 500 μm.

In addition to data from the analysis of a range of natural sheep scrapie, experimental sheep scrapie, experimental goat scrapie and BSE, derived agent strains, we also present data from the serial passage of CWD prions (derived from Mule Deer) in VM/Dk mice (prion agent strain 409V). Staining with thioflavin-S showed that amyloid PrP^d^ accumulations were a characteristic feature in the brain after infection with 409V prions (Figure 5A). In terminally affected VM mice these florid amyloid PrP plaques were mostly observed in the corpus callosum dorsal to the hippocampus and occasionally in the thalamus. In the brains of C57Bl/Dk mice large florid and multicentric plaques were observed in the corpus callosum and throughout the thalamus and hippocampus (Figure 5A). Comparison of the pattern of the PrP^d^ accumulation across all the prion agent strains used in this study suggested that the features of the amyloid accumulations could be reliably used to distinguish the CWD-derived 409V prion agent strain following experimental transmission to mice (Figures 3F, 5A).

### Glial Activation

Microglia are the phagocytic cells of the brain and their activation during CNS prion disease is also a prominent histopathological characteristic (Aguzzi and Zhu, 2017). Microglia are critical in host defence against prion disease and appear to phagocytose prions and delay disease pathogenesis (Carroll et al., 2018). Microglia proliferate and infiltrate prion-affected brain areas and display activated morphology as identified by immunostaining for allograft inflammatory factor 1 (AIF1; Figure 5B, upper row).

**FIGURE 6:**
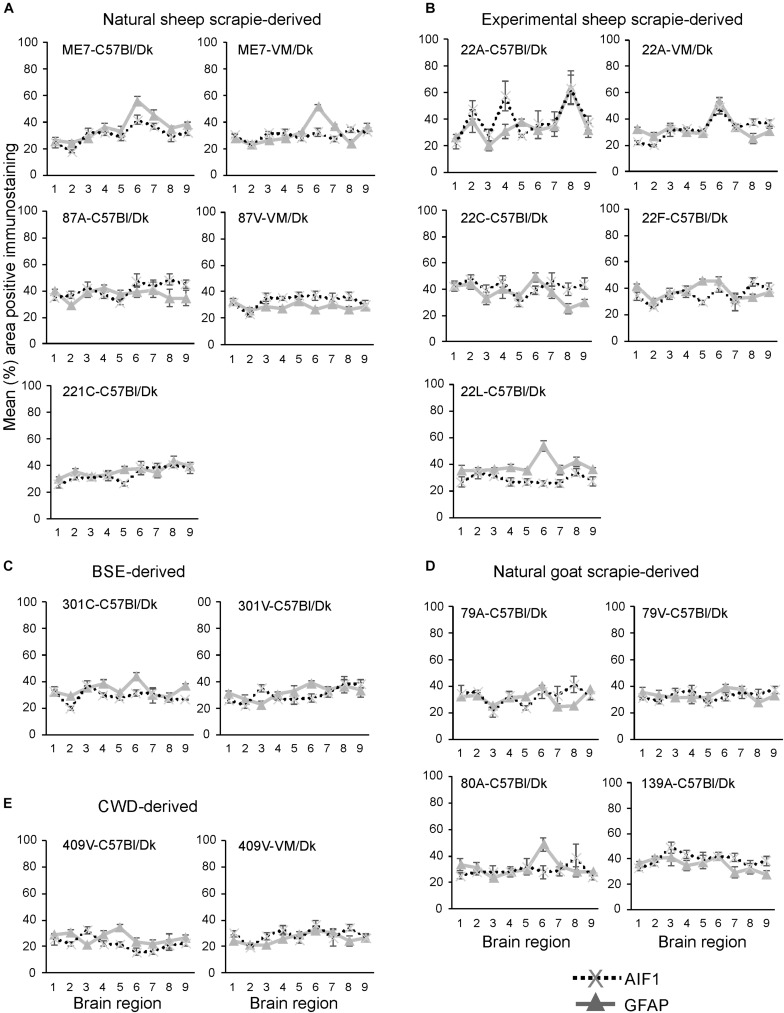
**(A–E)** Comparison of the magnitude and distribution of the GFAP+ and AIF1+ immunostaining in the brains of mice infected with a variety of distinct prion agent strains. Groups of C57Bl/Dk or VM/Dk mice were injected intracerebrally with a variety of distinct prion agent strains (Table 1). Brains were collected at the terminal stage of disease and immunostained to detect GFAP+ cells (astrocytes) and AIF1+ cells (microglia). The percentage area coverage of each marker was then compared in each of the following gray matter brain regions G1, dorsal medulla; G2, cerebellar cortex; G3, superior colliculus; G4, hypothalamus; G5, medial thalamus; G6, hippocampus; G7, septum; G8, cerebral cortex; G9, forebrain cerebral cortex. *N* = 6 mice/group. Data represent mean ± SE, from a minimum of 54 images/group.

Next, the percentage area coverage of AIF1 + immunostaining was measured across nine distinct brain gray matter areas (Figure 6). As above, the intensity of the AIF1 + immunostaining across these areas was then compared between each prion agent strain by paired *T*-test (Supplementary Table 2). This analysis showed the discriminatory extent of AIF1+ immunostaining in prion agent strain discrimination in the brain revealing statistically significant differences in 48% (73/153) of comparisons (Supplementary Table 2). Of note, prion agent strains with the highest levels (e.g., 22C-C57Bl/Dk and 87A-C57/Dk) and lowest levels (e.g., 409V-C57Bl/Dk) of AIF1+ immunostaining generated some of the highest statistically significant differences when compared to most other strains.

Astrocytes contribute to the maintenance of health and function of the CNS and are considered important in neurodegenerative diseases (Phatnani and Maniatis, 2015) including prion disease (Aguzzi and Liu, 2017). Reactive astrocytes were identified by immunostaining for the intermediate filament GFAP and their altered morphology (Figure 5B, lower row). The precise role and function of astrocytes in prion disease is not known beyond regional variance in their activation dependent upon prion strain (Monzon et al., 2018) and ability to replicate prions (Sarasa et al., 2012; Krejciova et al., 2017), hence their selection for further investigation in this study.

Next, the percentage area coverage of GFAP+ immunostaining was measured across nine distinct brain gray matter areas (Figure 6). The intensity of the GFAP+ immunostaining across these areas was then compared between each prion agent strain by paired *T*-test (Supplementary Table 3). This analysis suggested that GFAP+ immunostaining had the least discriminatory properties revealing statistically significant differences in only 34% (52/153) of prion agent strain comparisons. Of note, prion agent strains with low levels of GFAP+ immunostaining (e.g., CWD-derived 409V-C57Bl/Dk and 409V-VM/Dk) or large regional diversity (e.g., 22L-C57Bl/Dk and to a lesser extent 22F-C57Bl/Dk and 87V-VM/Dk) generated some of the highest statistically significant differences when compared to most other strains.

### CD44 Expression Is Upregulated During CNS Prion Disease

**FIGURE 7:**
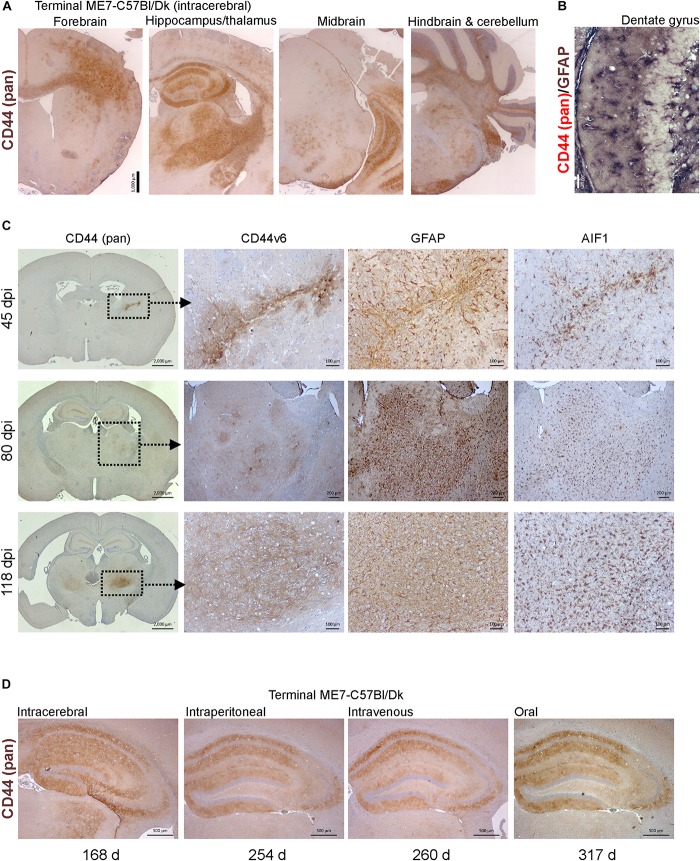
CD44 expression is upregulation in the brain during prion disease. **(A)** C57Bl/Dk mice were injected intracerebrally with ME7 scrapie prions and brains collected at the terminal stage of disease and immunostained to detect CD44. Scale bar = 1,000 μm. **(B)** Dual immunostaining for CD44 and GFAP suggested a correlation in their patterns implying an astrocyte origin. Scale bar = 100 μm. **(C)** C57Bl/Dk mice were injected intracerebrally with ME7 scrapie prions, brains were collected at intervals after infection and immunostained to detect CD44, CD44v6, GFAP and IBA1. Analysis of brains from 45, 80, and 118 days post injection (dpi) shows the progressive increase in expression from the needle-track, injection side thalamus and bilateral spread, respectively. Images are representative of *N* = 4 mice/group. Scale bars = 2,000, 100, or 200 μm as indicated. Upregulation of CD44 coincided with astrocyte (GFAP) and microglial (AIF1) responses. **(D)** CD44 pattern in terminal mouse brain following intracerebral, intraperitoneal, intravenous or oral ME7 infection reveals similar distribution of immunostaining irrespective of the route of exposure. Survival times (days) indicated. Images representative of *N* = 6 mice/group. Scale bars = 500 μm.

Molecular profiling of reactive astrocytes has identified CD44 as a pan-activation marker (Liddelow et al., 2017). In the brains of mice with terminal ME7 prion disease CD44 immunostaining was observed in a wide range of areas and sub-regions (Figure 7A). Furthermore, immunostaining for CD44 and GFAP suggested that CD44 was associated with astrocytes (Figure 7B).

Next, mice were injected IC with ME7 scrapie prions and the expression of CD44 in the brain compared throughout the pre-clinical phase. At 45 day post-injection (dpi) with prions, upregulated CD44 expression was only detected in association with the damage caused by the injection needle-track (Figure 7C, upper row). However, by 80 dpi CD44 expression was elevated in the thalamus on the same side of the brain as the injection site. This coincided with early signs of PrP^d^ accumulation, and evidence of astrocyte and microglia activation (Figure 7C, middle row). By 118 dpi CD44 upregulation was also observed in the contralateral thalamus and mirrored the distribution of the astrocyte activation (Figure 7C, bottom row). When *Cd44* mRNA is expressed alternative splicing can encode different CD44 isoforms. For example, CD44 variant 6 isoform (CD44v6) containing exon 11 is upregulated by and interacts with the chemotactic and phagocytosis inducing microglial expressed protein osteopontin (Weber et al., 1996; Katagiri et al., 1999; Gao et al., 2003; Marroquin et al., 2004; Khan et al., 2005; Morisaki et al., 2016). Our data suggest that the CD44 expressed during these early pre-clinical stages appeared to be predominantly of the CD44v6 isoform (Figure 7C).

We next determined whether the route of prion exposure influenced the expression of CD44 in the brain. Our analysis showed a similar upregulation of CD44 expression in the brain at the terminal stage of disease after exposure to prions via intraperitoneal injection, IV injection or oral infection (Figure 7D). These data show that for the ME7 prion agent strain the specified pattern of CD44 upregulation in the CNS during prion disease is unaffected by the route of infection.

### Distinct Patterns of CD44 Expression in the Brains of Mice Infected With Different Prion Agent Strains

**FIGURE 8:**
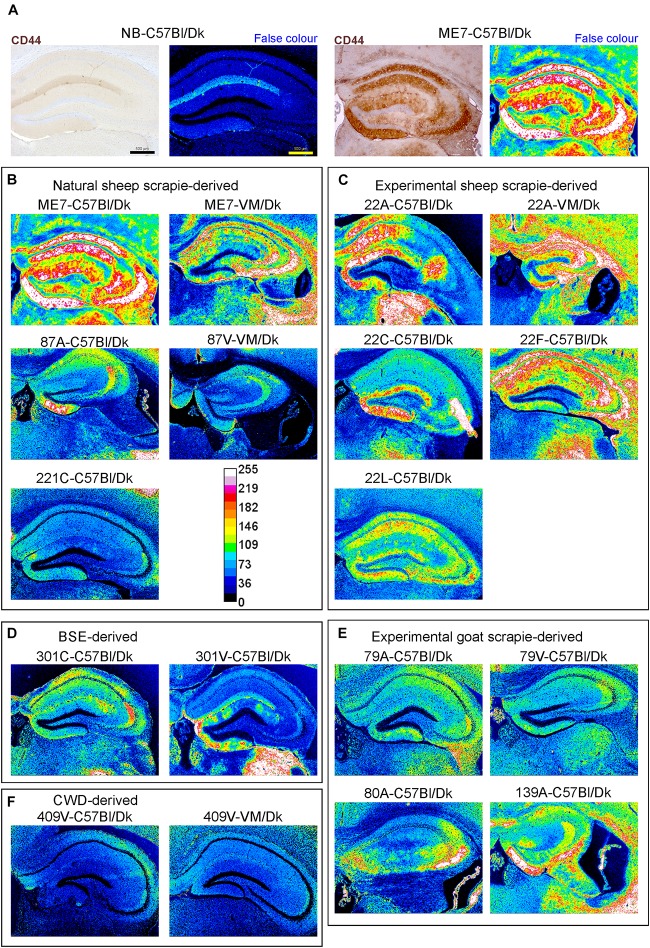
Distinct patterns of CD44+ immunostaining in the hippocampus region of the brains of mice infected with a variety of distinct prion agent strains. Groups of C57Bl/Dk or VM/Dk mice were injected intracerebrally with a variety of distinct prion agent strains (Table 1). Brains were collected at the terminal stage of disease and immunostained to detect CD44. Images were then false-colored to compare the signal intensities of the immunostaining between each group. **(A)** Normal brain infected mice display little CD44+ immunostaining when compared to mice terminally affected with ME7 scrapie prions. **(B–F)** Comparison of false colored images to show the variation in CD44+ immunostaining between diverse prion strains isolated from a variety of sources. Representative images from 6 mice/group are shown. Scale bars = 500 μm.

Little if any CD44 expression was detected by IHC in the brains of uninfected control mice, whereas abundant CD44 expression was detected in the brains of C57Bl/Dk mice infected with ME7 scrapie prions (Figure 8A). We next compared the distribution and abundance of CD44 expression in the brains of mice infected with distinct prion agent strains. To specifically compare the abundance and variance of the CD44 upregulation between prion agent strains, immunostained images were false-colored in respect to pixel intensity. This analysis revealed patterns of CD44 upregulation in the brain that were specific for each prion agent strain (Figures 8B–E). Furthermore, by comparing the CD44 expression profile specifically within the hippocampus, it was possible to discriminate each prion agent strain/host combination based solely on CD44 localization (Table 2 and Figure 8). CD44 heterogeneity within activated astrocytes has been previously identified in mouse models of Alexander disease (Sosunov et al., 2013). Our data suggest that CD44 upregulation within the hippocampus is also a useful marker to discriminate the neuropathological changes during CNS prion disease.

**TABLE 2:** Prion agent strain typing based on hippocampal CD44 expression pattern.

**Strain-Host**	**CA1**	**CA2**	**CA3**	**DG**
ME7-C57/Dk	+++ so +++ sr + slm	+ so + sr	+++ so + sp ++ slu +++ sr	++ po ++ superior mo +++ inferior mo
22A-C57/Dk	+++ so^∗^ +++ sr^∗^ +++ slm^∗^	–	+++ sr^∗^	+++ superior mo^∗^ ++ inferior mo^∗^
22C-C57/Dk	+ so	+ so	++ sr^∗^ ++ so^∗^	++ superior mo^∗^ +++ inferior mo^∗^
22F-C57/Dk	+++ so^∗^ ++ sp +++ sr^∗^ +++ slm^∗^	++ so^∗^ ++ sp ++ sr^∗^	++ so^∗^ ++ sr^∗^	++ superior mo + inferior mo ^∗^mo apex
4 22L-C57/Dk	+ so^∗^ ++ sr^∗^	+ so^∗^ + sr^∗^	++ so^∗^ + slu + sr	+ superior mo ++ inferior mo
79A-C57/Dk	+ so + sr	+ so + sr	++ so ++ slu	+ mo ‘astrocytic’
79V-C57/Dk	–	+ so + sr	+++so ++ slu + sr + slm	+ inferior mo
80A-C57/Dk	–	+ so	+slu +++ so	+++ inferior mo
87A-C57/Dk	+ so + sr	+ so +++ sr	+ slu	++ inferior mo^∗^
139A-C57/Dk	++ so ++ sr	+ so + sr	+++ so ++ slu	+++ inferior mo
221C-C57/Dk	–	–	++ so + slu	+ ‘astrocytic’/inferior mo
409V-C57/Dk	–	–	–	+ peri plaque DG/Thalamus
301C-C57/Dk	+ so^∗^	+ so^∗^ + sr ++ slm^∗^	+ slu	–
301V-C57/Dk	+ slm	–	+ slu	+++ mo ‘astrocytic’
ME7-VM/Dk	++ so^∗^ ++ sp ++ sr^∗^ ++ slm	++ so ++ sr ++ slm	+++ so^∗^ +++ sr^∗^ + slm	+ mo ‘astrocytic’ + po
22A-VM/Dk	+++ so^∗^ ++ sp +++ sr^∗^ +++ slm^∗^	++ so^∗^ ++ sr^∗^ +++ slm^∗^	++ so^∗^ +++ sr^∗^ +++ slm	++ mo^∗^ ++ po^∗^
87V-VM/Dk	+ so + sr ++ slm	+ so + sr	+ so ++ slu	++ apex mo
409V-VM/Dk	+ peri plaque so/cc	–	–	–

*^∗^Regions also stain positive for CD44v6. cc, corpus callosum; mo, molecular layer; po, polymorph layer; slm, stratum lacunosum moleculare; slu, stratum lucidum; so, stratum oriens; sp, stratum pyrimidale; sr, stratum radiatum.*

This analysis also revealed that the abundance of the CD44 expression in the brains of mice with terminal prion disease appeared to mirror the magnitude and distribution of the PrP^d^. For example, in prion agent strain/recipient mouse genotype combinations that displayed diffuse PrP^d^ accumulation e.g., ME7, experimental sheep scrapie 22A, 22C, 22F, and 22L and experimental goat scrapie derived strains 79A, 79V, 80A and 139A, the abundance of the CD44 expression was similarly diffuse (Figures 3B,C,E, 8B,C,E). However, in mice infected with 409V prions, the CD44 expression almost exclusively associated with regions containing amyloid PrP^d^ accumulations.

Since host *Prnp* genotype can significantly affect prion disease survival times and the characteristics of the neuropathology (Dickinson and Fraser, 1977; van Keulen et al., 2015), we next compared CD44 expression in the brains of C57Bl/Dk mice or VM/Dk mice infected with the ME7 or 22A scrapie prion agent strains. This analysis showed infection of C57Bl/Dk mice or VM/Dk mice with the same prion agent strain gave rise to distinct patterns of CD44 expression in the hippocampus (Figures 8B,C). This analysis suggests that host *Prnp* genotype may also influences the expression pattern of CD44 in the CNS during prion disease.

**FIGURE 9:**
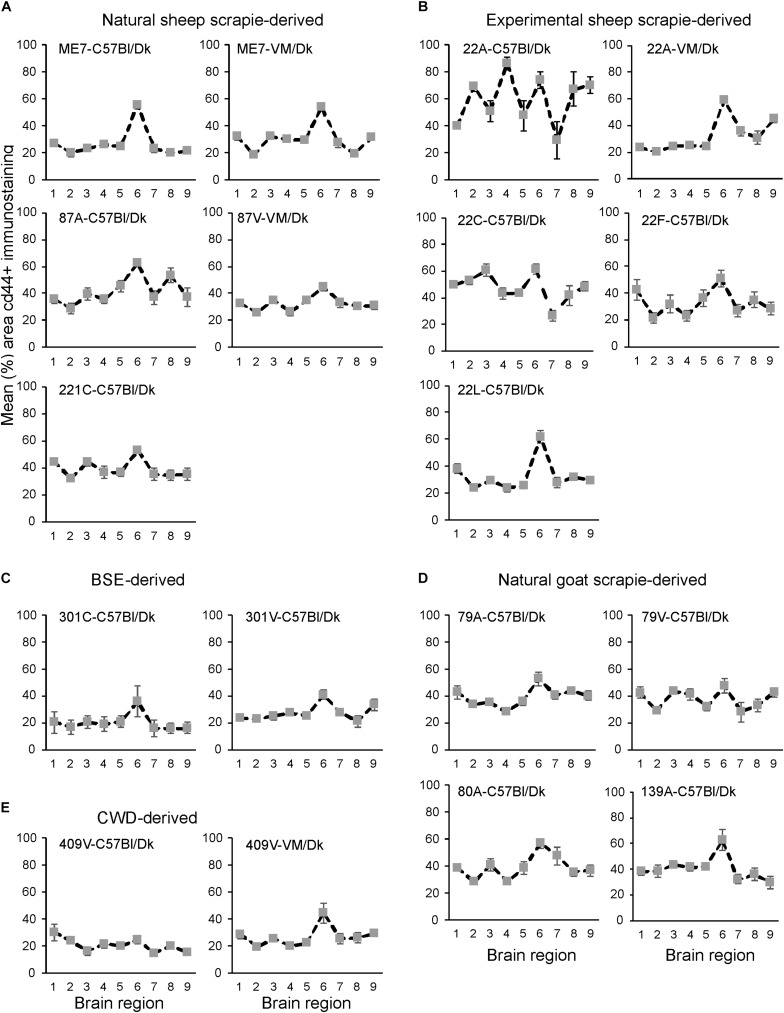
**(A–E)** Comparison of the magnitude and distribution of the CD44+ immunostaining in the brains of mice infected with a variety of distinct prion agent strains. Groups of C57Bl/Dk or VM/Dk mice were injected intracerebrally with a variety of distinct prion agent strains (Table 1). Brains were collected at the terminal stage of disease and immunostained to detect CD44. The percentage area coverage of CD44+ immunostaining was then compared in each of the following gray matter brain regions G1, dorsal medulla; G2, cerebellar cortex; G3, superior colliculus; G4, hypothalamus; G5, medial thalamus; G6, hippocampus; G7, septum; G8, cerebral cortex; G9, forebrain cerebral cortex. *N* = 6 mice/group. Data represent mean ± SEM, from a minimum of 54 images/group.

Next, the magnitude of the CD44+ immunostaining was compared for each strain/host combination investigated by calculating the mean percentage area coverage across nine distinct gray matter areas (Figures 9A–E). The intensity of the CD44+ immunostaining across these areas was then compared between each prion agent strain by paired *T*-test (Supplementary Table 4). This analysis highlighted the discriminatory nature of reactive astrocyte heterogeneity in CD44 in the brains of mice infected with distinct prion agent strains. Furthermore, this analysis suggested that CD44 + immunostaining had the most discriminatory properties revealing statistically significant differences in 70% (107/153) of comparisons. Of note BSE (301C and 301V) and CWD (409V) derived strains and ME7-C57Bl/Dk were highly statistically significantly different from other scrapie derived strains. Furthermore the same prion strain, e.g., ME7 or 22A produced statistically significantly different profiles when in different *Prnp* genotype hosts.

Representative images of AIF1+; CD44+, CD44v6+, GFAP+ and PrP^d^+ immunostaining on serial sections from brains from all the prion agent strain/recipient mouse combinations studied are provided in Supplementary Figure 1. Analysis of the correlation of these neuropathological markers of CNS prions disease (AIF1 + microglia; CD44+ and GFAP+ astrocytes; PrP^d^) showed the variance across all the prion agent strain/recipient mouse combinations studied (Supplementary Table 5). This analysis suggested that the strongest and most significant correlate was CD44 with GFAP amongst most strain-host combinations. Also of note was the lack of correlation with AIF1+ microglia when comparing the neuropathology of ME7 prion agent strain in the brains of C57Bl/Dk mice and VM/Dk mice.

### CD44 Upregulation Is Astrocytic and Correlates With Prion Accumulation

**FIGURE 10:**
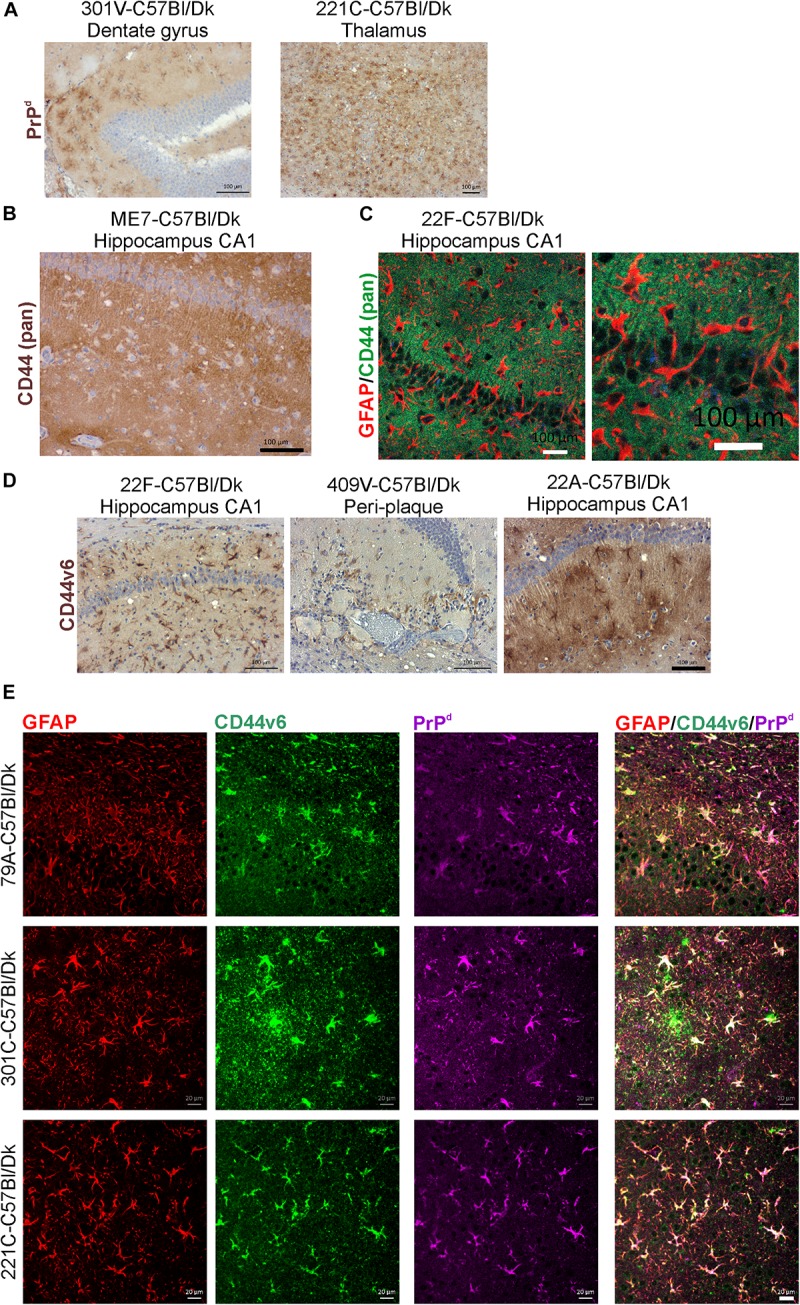
CD44v6 expression and PrP^d^ colocalize with GFAP + astrocytes in the brains during prion disease. **(A)** Immunohistochemical demonstration of astrocytic PrP^d^ immunostaining patterns in brains of mice infected with certain prion agent strains. **(B,C)** Immunohistochemical analysis shows pan-CD44 expression surrounds but does not co-localize with GFAP in astrocytes. **(D)** Immunostaining for CD44v6 shows astrocytic staining pattern suggesting that astrocytes are the predominant cellular source of CD44 upregulation during prion disease. **A–C** scale bars = 100 μm. **(E)** Immunofluorescent analysis of CD44, GFAP and PrP^d^ in the brains of mice with prion disease shows that CD44+ /GFAP+ astrocytes accumulate PrP^d^. Scale bars = 20 μm.

In the brains of mice infected with several prion agent strains, astrocytic patterns of PrP^d^ deposition can be detected (Figure 10A). Our analysis suggested that the regions of the brain with astrocytic prion accumulation mirrored those displaying high levels of CD44. Immunostaining with the N-terminal pan-CD44 antibody showed diffuse neuropil staining with un- or lightly stained astrocytes in relief (Figure 10B). Co-staining with the pan-CD44 antibody and GFAP similarly showed minimal direct co-localisation between these two proteins in astrocytes (Figure 10C). This observation is consistent with the demonstration that the pan-CD44 antibody recognizes an N-terminal epitope in the cleavable and soluble ectodomain of the CD44 protein (Nagano and Saya, 2004).

Activation of CD44v6 isoform on endothelial cells requires association of the CD44 carboxy-terminus with ezrin that couples the CD44v6 isoform to the cytoskeleton (Tremmel et al., 2009). At the terminal stage prion disease our IHC analysis showed that the CD44v6 isoform displayed either an intense staining pattern throughout the astrocyte, or mixed pattern including strong staining throughout the astrocyte and a surrounding diffuse staining pattern (Figure 10D). The pattern of CD44v6 immunostaining appeared to be more distinctly localized when compared to the widespread and diffuse pattern obtained with pan-CD44 staining. It is plausible that the pan-CD44 upregulation observed at the terminal stage also includes standard CD44 and/or splice variants other than CD44v6. For example, in the brains of human Alzheimer’s disease patients upregulation of splice variants CD44v3 and CD44v10 as well as CD44v6 has also been observed (Pinner et al., 2017). This combination of staining patterns also suggests that CD44v6 may have undergone extracellular cleavage and binding to the cytoskeleton in specific astrocyte subpopulations (Figures 10D,E). IHC for GFAP, CD44v6 and PrP^d^ revealed colocalization in specific astrocyte populations (Figure 10E).

## Discussion

Prion diseases are a diverse group of infectious neurodegenerative disorders that are considered to be caused by abnormally folded prion protein. The observation of different clinical syndromes following the transmission of experimental scrapie prions in goats led to the suggestion of variation in prion strains (Pattison and Millson, 1961). Transmissions into laboratory mice proved that prion disease isolates may be resolved into individual strains of infectious agent, that retain specific properties including the duration of the survival time and the targeting of the neuropathology to defined brain areas (Bruce, 2003). Of course, one of the major contentious issues in the prion hypothesis is how a misfolded host protein (PrP^Sc^) can encode such diverse strain-specific properties. Plausibly, the prion agent diversity may be encoded partly or in combination through variation in primary structure, N-linked glycosylation and folding confirmation (Morales et al., 2007). The exact mechanisms that control prion agent strain targeting within the CNS are unknown, despite the neuroinflammatory nature of prion neurodegeneration being identified (Burwinkel et al., 2004; Riemer et al., 2009; Crespo et al., 2012).

In this study, we set out to characterize astrocyte responses to prion infection and uncovered a host response to infection that defines prion strain targeting within the CNS. Until now prion strain typing has typically involved lengthy and expensive transmission experiments in panels of laboratory mice (Boyle et al., 2017). However, this detailed prion strain-typing is necessary for the identification and characterization of novel and emerging prion diseases, especially those with zoonotic potential. Here we show that prion agent strains can be readily discriminated via profiling of astrocyte CD44 expression within the CNS. In the current study, astrocyte responses to prion infection were characterized using anti-pan-CD44 and the splice variant specific anti-CD44v6 antibodies on a wide-range of mouse-adapted prion disease strains derived from sheep scrapie, BSE and CWD. We show that the upregulation of CD44 expression occurs in sub-region specific brain areas revealing an undiscovered level of heterogeneity in astrocyte populations following prion infection. Further analysis showed that the variety in the presentation of the CD44+ immunostaining between each prion stain/host combination revealed unique and distinguishable patterns, especially within the hippocampus. Indeed even in the brains of mice infected with the amyloidogenic mouse-adapted CWD strain 409V, the astrocytes surrounding the PrP plaques responded via upregulating both GFAP and CD44. Furthermore, the expression profile of the CD44+ immunostaining in mice infected with the ME7 scrapie agent strain was unaltered by the route of infection. CD44 was upregulated as early as halfway through the incubation period and occurred concurrently with some of the earliest neuropathological changes. CD44 positive astrocytes also displayed accumulation of PrP^Sc^, suggesting they may act as reservoirs for prion production within the CNS during infection.

Following on from our analysis of prion agent strain-specific CD44 patterns in brains of experimentally infected mice, further work is now required to investigate the variation in CD44 expression patterns in natural prion disease in both human and animal hosts. Such analysis is essential to determine whether this the pattern of the CD44+ immunostaining in the brain can similarly be used to discriminate distinct prion diseases in these natural host species.

The pattern and morphology of the pan-CD44 labeling in prion-infected brain indicated an astrocytic source, as observed previously in Alzheimer’s disease (Akiyama et al., 1993). Immunostaining of prion disease-affected brains with an antibody against the common (pan) extracellular N-terminus of CD44 surrounded, but did not co-localize, with cytoplasmic and cytoskeletal GFAP. To provide additional confirmation that the source of the CD44 was astrocytic in nature we investigated further the expression of splice variant CD44v6, since independent studies have shown that when this variant is upregulated in certain cell populations it is coupled to the cytoskeleton when activated (Tremmel et al., 2009). Our data suggested that the distribution of splice variant CD44v6 expression was mostly associated with the GFAP+ compartment of relevant reactive astrocytes following prion infection. In some strain/host combinations, additional diffuse immunostaining was also apparent in association with the cell membrane. Furthermore, the lack of labeling with the N-terminal pan-CD44 antibody in that same GFAP+ compartment implies that the v6 epitope in the extracellular stem region of the CD44 protein is retained whereas the panCD44 N-terminal epitope is not. Thus it is plausible that during CNS prion disease intracellular and potentially activated CD44v6 has been N-terminally cleaved by extracellular proteases, rather than intracellular γ-secretase cleavage (Nagano and Saya, 2004; Pelletier et al., 2006; Anderegg et al., 2009). Data from Alzheimer’s disease patients has also revealed increases in other CD44 splice variants, in particular CD44v3 and CD44v10 (Pinner et al., 2017). Whether the expression of other CD44 splice variants is similarly induced in astrocytes during prion disease remains to be determined.

Together, our data show that during CNS prion disease specific reactive astrocytes express high levels of CD44 and the splice variant form, CD44v6. Analysis of CD44 expression in distinct regions throughout the brain also suggested that CD44 was not universally upregulated in GFAP+ activated astrocytes. The precise role of the CD44+ astrocytes during CNS prion disease remains to be determined. CD44 is a multifunctional single-chain transmembrane glycoprotein, which mediates cellular responses to the extracellular environment (Ponta et al., 2003; Dzwonek and Wilczynski, 2015). Within the CNS CD44 has been shown to regulate the functional and structural plasticity and integrity of dendritic spines and synapses (Skupien et al., 2014; Roszkowska et al., 2016), as well as regulating astrocyte morphology (Konopka et al., 2016). Since these features are adversely affected during CNS prion disease (Jeffrey et al., 1995, 2000) it is plausible that the altered CD44 expression described here may contribute to the development of the neuropathology. Transcriptomic analyses of the reactive astrocytes induced by various pro-inflammatory stimuli have shown that CD44 expression is significantly elevated in neurotoxic A1 astrocytes when compared to A2 astrocytes (Liddelow et al., 2017). Thus is it plausible that the enhanced expression of CD44 or CD44v6 during CNS prion disease may also be indicative of a neurotoxic phenotype in the reactive astrocytes. Specific blockade of the ability of microglia to stimulate A1 astrocyte conversion can provide neuroprotection in a mouse model of α-synucleinopathy-induced neurodegeneration (Yun et al., 2018). Further studies are required to determine whether the modulation of astrocyte activation may likewise be beneficial during CNS prion disease.

## Data Availability

All datasets generated for this study are included in the manuscript and/or the Supplementary Files.

## Ethics Statement

The animal studies were reviewed and approved by the Institute for Animal Health Neuropathogenesis Unit Ethical Review Committee, the University of Edinburgh Ethical Review Committee, and the UK Home Office.

## Author Contributions

BB and NM contributed to the conception and design of the study, and wrote the manuscript. BB and CW acquired the data and performed the statistical analysis. All authors interpreted the data and contributed to the final version of the manuscript revision.

## Conflict of Interest Statement

The authors declare that the research was conducted in the absence of any commercial or financial relationships that could be construed as a potential conflict of interest.

## Footnotes

1 http://rsb.info.nih.gov/ij/
