# Supplementary material for: Discrimination of Prion Strain Targeting in the Central Nervous System via Reactive Astrocyte Heterogeneity in CD44 Expression
Source: Front Cell Neurosci. 2019 Sep 10;13:411. doi: 10.3389/fncel.2019.00411 (PMC6746926; doi:10.3389/fncel.2019.00411)
Supplement: Supplementary file 1 [file Data_Sheet_1.PDF]

Supplementary Figure 1. Example images of PrP^d+, GFAP+, IBA1+, CD44+ and CD44v6+ immunostaining in the brains of mice infected with a range of prion agent strains. Groups of C57Bl/Dk mice and VM/Dk mice were injected intracerebrally with distinct prion agent strains from a range of sources (see Table 1). At the terminal stage of disease brains were collected and immunostained to detect PrP^d, GFAP, AIF1, CD44 and CD44v6. For each prion agent strain and mouse host combination data from a series of serial immunostained images from the brain of one mouse from each group are shown. False coloured images show the relative expression intensities of the PrPd+ and CD44+ immunostaining.

Natural sheep scrapie derived

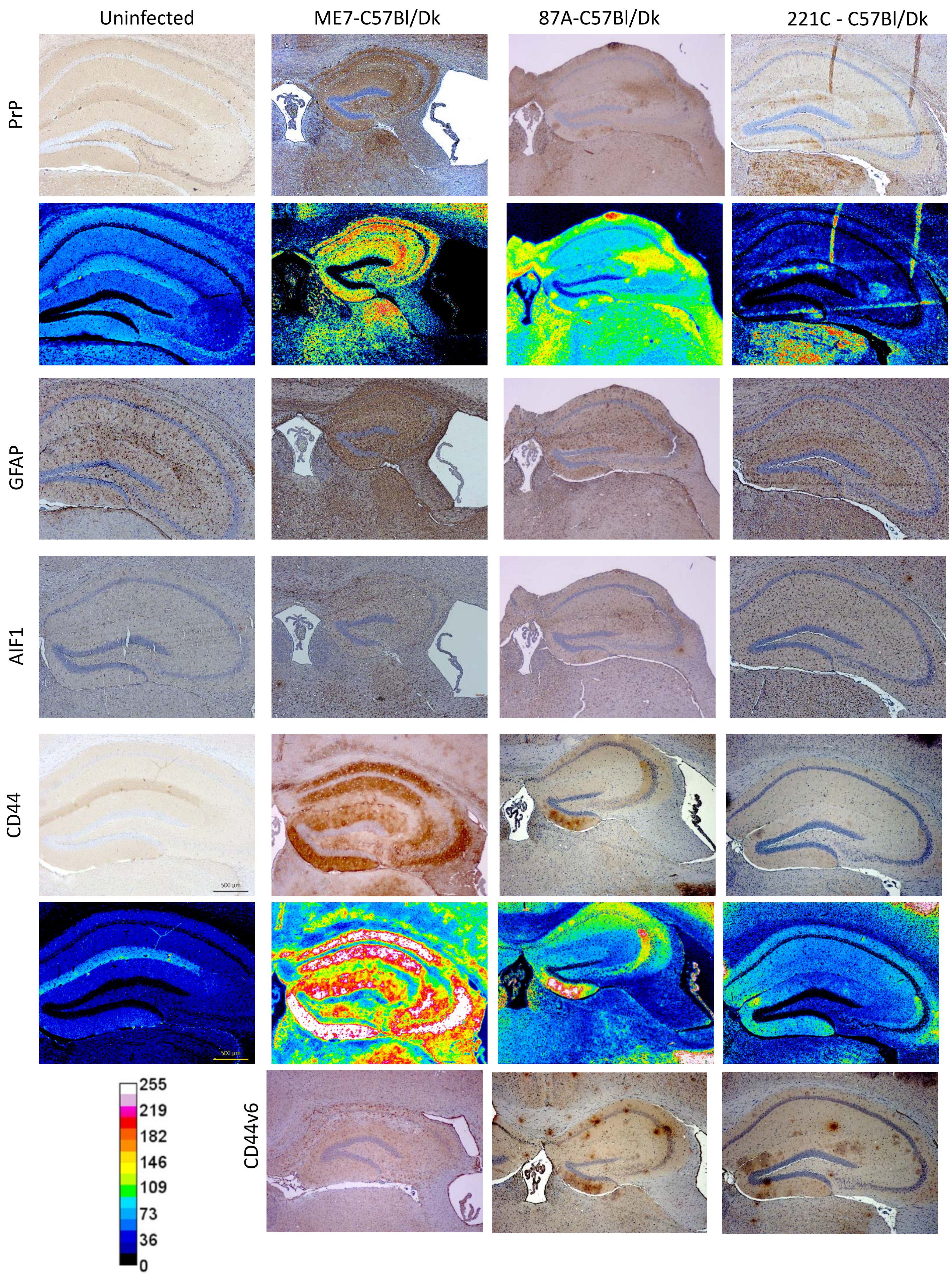

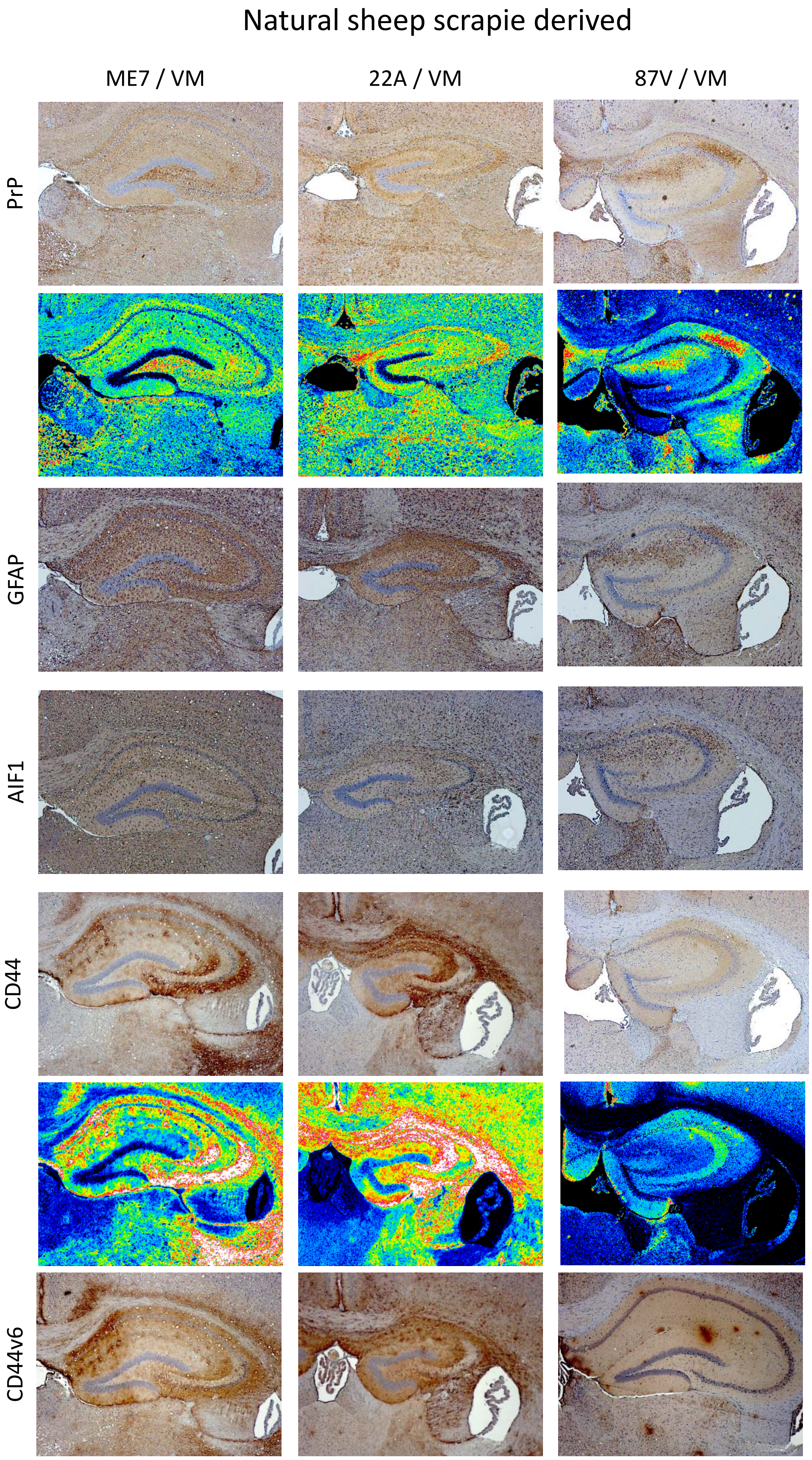
Natural sheep scrapie derived
ME7 / VM
22A / VM
87V / VM
PrP
GFAP
AIF1
CD44
CD44v6

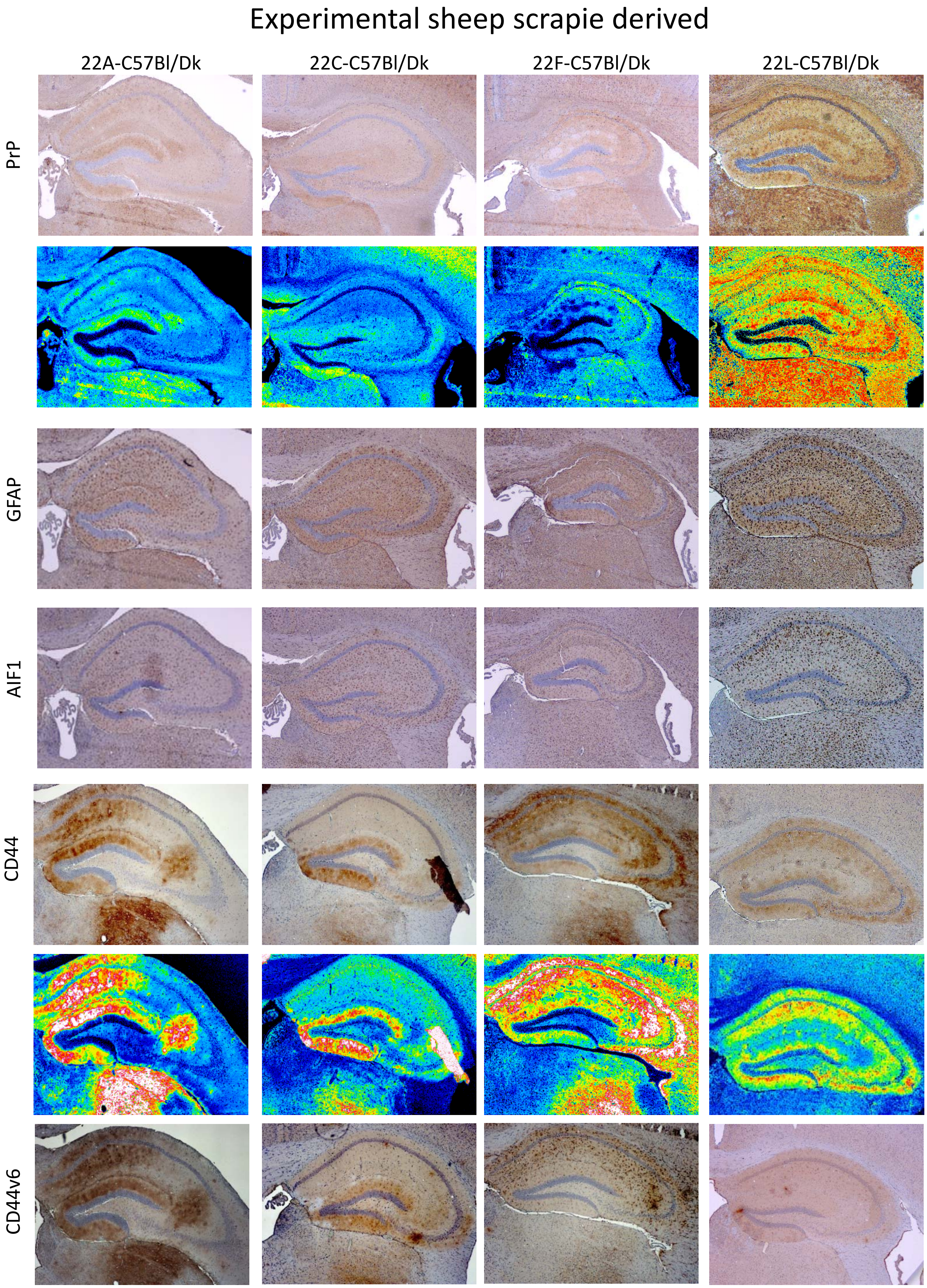
Experimental sheep scrapie derived
22A-C57Bl/Dk
22C-C57Bl/Dk
22F-C57Bl/Dk
22L-C57Bl/Dk
PrP
GFAP
AIF1
CD44
CD44v6

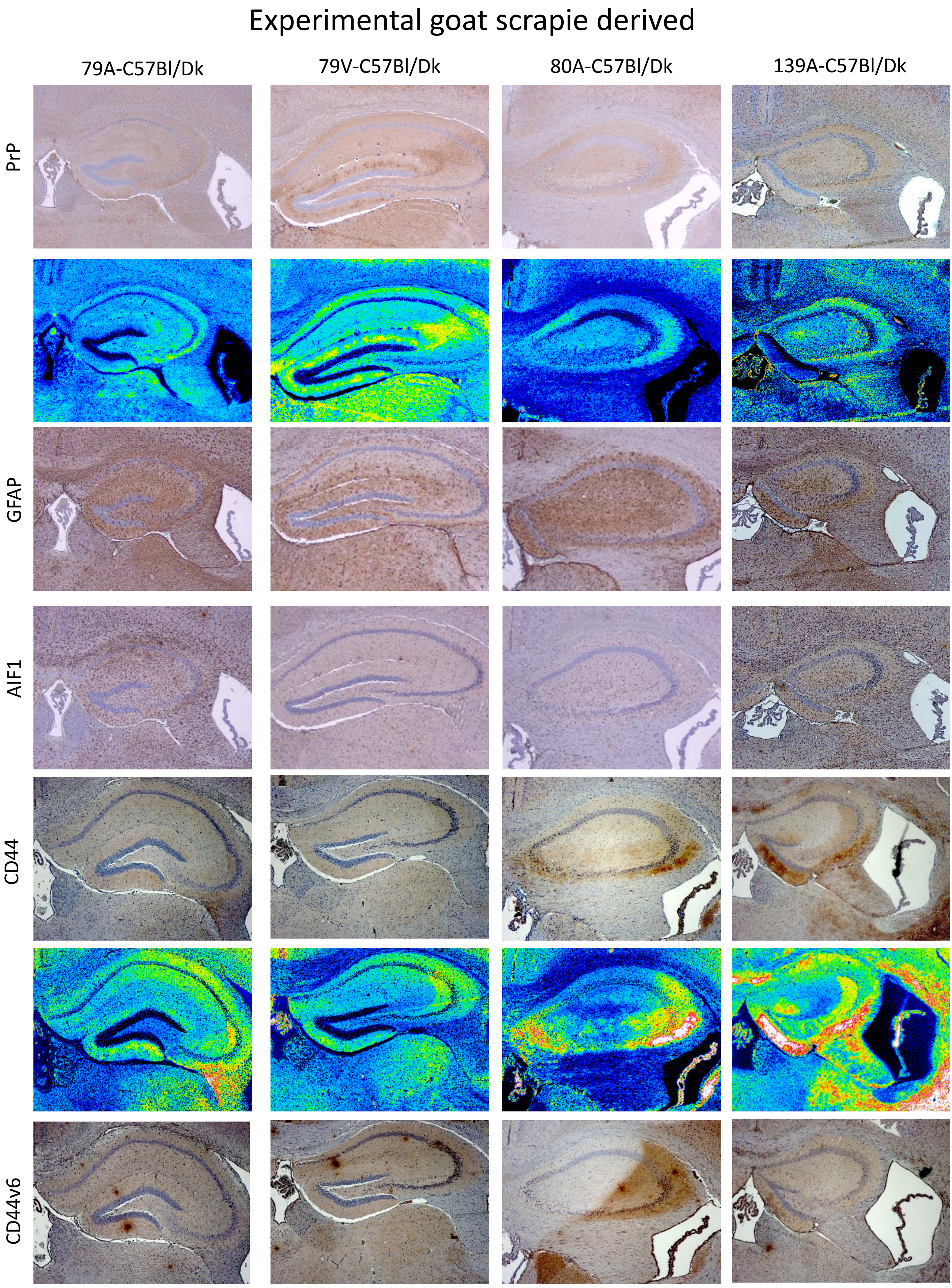
Experimental goat scrapie derived
79A-C57Bl/Dk
79V-C57Bl/Dk
80A-C57Bl/Dk
139A-C57Bl/Dk
PrP
GFAP
AIF1
CD44
CD44v6

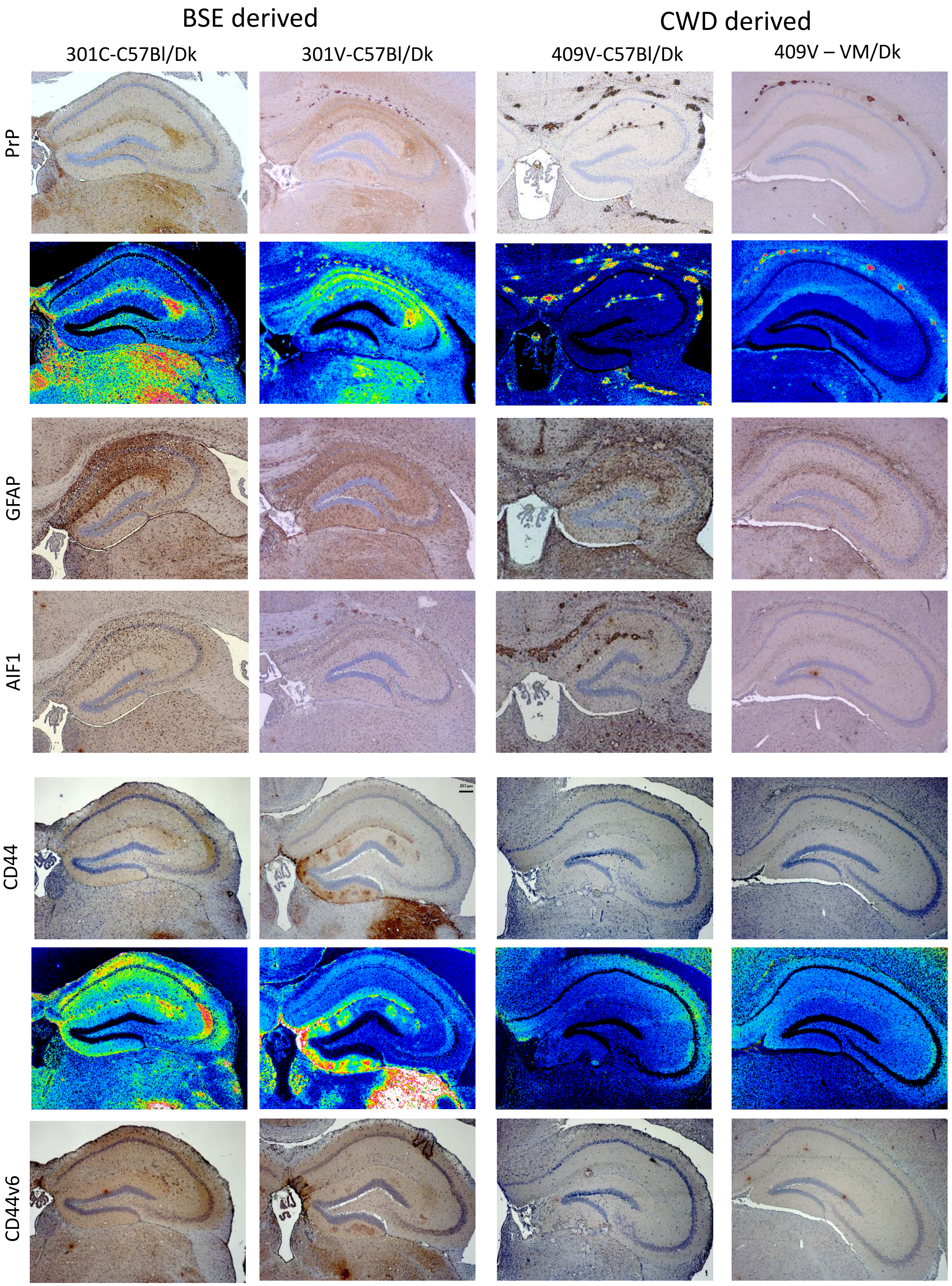
BSE derived
CWD derived
301C-C57Bl/Dk
301V-C57Bl/Dk
409V-C57Bl/Dk
409V – VM/Dk
PrP
GFAP
AIF1
CD44
CD44v6
